# Supplementary material for: Thermoplastic Starch Composites Reinforced with Functionalized POSS: Fabrication, Characterization, and Evolution of Mechanical, Thermal and Biological Activities
Source: Antibiotics (Basel). 2022 Oct 17;11(10):1425. doi: 10.3390/antibiotics11101425 (PMC9598116; doi:10.3390/antibiotics11101425)
Supplement: Supplementary file 1 [file antibiotics-11-01425-s001.zip › antibiotics-1928681-supplementary.pdf]

Supplementary data

Table S1. Thermogravimetric analysis of TS and TS/*fn*-POSS nanocomposite films.

Samples	Initial Degradation Temperature, °C	Second Degradation Temperature, °C	Final Degradation Temperature, °C	Ash Content (%)
TSP-0.0	220.51	382.10	395.35	6.20
TSP-0.5	225.60	390.25	401.50	5.91
TSP-1.0	247.92	406.82	414.15	5.85
TSP-3.0	275.65	427.35	435.82	5.24
TSP-5.0	350.58	460.54	469.50	4.75

Table S2. Values of thickness, water solubility (WS), water vapor transmission rate (WVTR), oxygen transmission rate (OTR) and water contact angle (WCA of the TS and TS/*fn*-POSS nanocomposite samples.

Properties	TS/*fn*-POSS Nanocomposite Samples				
	TSP-0.0	TSP-0.5	TSP-1.0	TSP-3.0	TSP-5.0
Thickness (µm)	54 ± 0.02[a]	59 ± 0.03[b]	65 ± 0.07[b]	78 ± 0.10[a]	91 ± 0.05[c]
Water solubility (%)	78.20	70.55	59.85	37.35	25.11
WVTR (g/m²/day)	72.4	69.0	64.9	50.8	48.1
OTR (cc/m² /24 h)	140.2	128.9	107.1	78.3	51.8
WCAº (%)	56.4	57.8	60.5	64.1	76.3

Note: Values with the same superscript letter in the same row indicate that they are not statistically different ($p < 0.05$). The values are presented as mean ± SD.

Table S3. Antimicrobial activity of TS/*fn*-POSS nanocomposites against *S. aureus* and *E. coli.*

Strain	Zone of Inhibition in (mm)				
	TSP-0.0	TSP-0.5	TSP-1.0	TSP-3.0	TSP-5.0
S. aureus	11.0	11.9	13.7	13.9	15.0
E. coli	11.0	11.3	13.2	13.7	14.8
